# Comparison of the Hospital Arrival Time and Differences in Pain Quality between Diabetic and Non-Diabetic STEMI Patients

**DOI:** 10.3390/ijerph120201387

**Published:** 2015-01-27

**Authors:** Marina Gradišer, Dario Dilber, Jasna Cmrečnjak, Branko Ostrički, Ines Bilić-Ćurčić

**Affiliations:** 1 Department of Endocrinology and Diabetes, County Hospital Čakovec, 40000 Čakovec, Croatia; E-Mail: gradiser007@yahoo.com; 2 Department of Cardiology, County Hospital Čakovec, 40000 Čakovec, Croatia; E-Mails: dario.dilber@gmail.com (D.D.); jcmrec@gmail.com (J.C.); branko.ostricki@vz.t-com.hr (B.O.); 3 Clinical Department of Diabetes, Endocrinology and Metabolism Disorders, University Hospital Centre Osijek, 31000 Osijek, Croatia; 4 Faculty of Medicine, Josip Juraj Strossmayer University of Osijek, 31000 Osijek, Croatia

**Keywords:** STEMI, diabetes type 2, quality of pain, time of arrival, outcome, prognosis

## Abstract

The aim of our study was to determine whether diabetic ST segment elevation myocardial infarction (STEMI) patients arrive in the emergency room (ER) later than non-diabetics, compare the differences in pain quality and quantity between those groups, and measure differences in the outcome after an index hospitalization. A total of 266 patients with first presentation of STEMI were included in our study during a period of two years, 62 with diabetes and 204 without diabetes type 2. Pain intensity and quality at admission were measured using a McGill short form questionnaire. Diabetic patients did not arrive significantly later than non-diabetic (χ^2^; *p* = 0.105). Most diabetic patients described their pain as “slight” or “none” (χ^2^; *p* < 0.01), while most non-diabetic patients graded their pain as “moderate” or “severe” (χ^2^; *p* < 0.01). The quality of pain tended to be more distinct in non-diabetic patients, while diabetic patients reported mainly shortness of breath (χ^2^; *p* < 0.01). Diabetic patients were more likely to suffer a multi-vessel disease (χ^2^; *p* < 0.01), especially in the late arrival group. Therefore, cautious evaluation of diabetic patients and adequate education of target population could improve overall survival while well-organized care like a primary PCI Network program could significantly reduce CV mortality.

## 1. Introduction

Cardiovascular disease, particularly coronary artery disease (CAD), is one of the leading causes of morbidity and mortality in patients with diabetes [1]. In those patients, clinical presentation of CAD can be altered, atypical or absent, which can delay diagnosis and appropriate treatment. Diabetic patients without a history of CAD can have the same overall cardiac risk as non-diabetic patients who already suffered myocardial infarction (MI) [2]. The presence of diabetes is a strong, independent predictor of short-term and long-term recurrent ischemic events, including mortality [3,4,5,6]. A negative impact of diabetes on outcomes in patients with the acute coronary syndrome (ACS) is present in both, the acute phase and during the post-ACS follow up [7,8,9].

Diabetes has a major impact on CAD development and its consequences, autonomic neuropathy, and diabetic cardiomyopathy [3]. It has been estimated that about 20% of asymptomatic diabetic patients have an abnormal cardiovascular autonomic function [8]. The risk of cardiovascular autonomic neuropathy depends on duration of diabetes and on degree of glycemia dysregulation [3,8]. “Silent ischemia” refers to the presence of objective findings suggestive of myocardial ischemia without angina or anginal equivalent symptoms [10]. Diabetic patients often present with the atypical presentation of stable angina, as well as in patients with acute forms of CAD, *i.e.*, unstable angina and myocardial infarction. That atypical presentation of ACS, more frequent in diabetic patients, includes also some other anginal equivalents such as nausea, vomiting, diaphoresis, epigastric discomfort, dyspnea and an unexplained fatigue [10].

The purpose of our study was to assess whether diabetic patients with STEMI arrive in the emergency room (ER) later than non-diabetic patients, and whether possible differences in pain quality between those two groups could influence the arrival time to the emergency department which could be explained by diabetic polyneuropathy leading to the impaired symptom perception and causing worse clinical outcome. To confirm this hypothesis, we analyzed the hospital arrival time (from onset of pain to arrival to the ER), the chest pain intensity and other potential symptoms’ differences between two groups. Furthermore, a comparison has been done according to survival, results of PCI, peak troponin T levels (cTnT) and duration of hospitalization.

## 2. Results

From the total of 266 patients, 204 (76.7%) were non-diabetic patients and 62 (23.3%) were diabetic patients (Table 1). Men with diabetes were significantly younger than their counterparts without diabetes (*u*-test, *p* = 0.013). There was no such difference for women (*u*-test, *p* = 0.774). The overall significance of age difference between diabetic and non-diabetic group was present due to the greater number of men (*u*-test; *p* = 0.049). There was no significant difference among groups with respect to sex distribution, smoking status, alcohol consumption, blood pressure, lipid profiles, renal function or use of aspirin, statins and antihypertensive drugs (data not shown).

**Table 1:** Baseline characteristics of subjects and arrival time in diabetic and non-diabetic group.

Baseline Characteristics and Arrival Time of Subjects	Non-Diabetic (*N* = 204, 76.7% of total)	Diabetic (*N* = 62, 23.3% of total)	*p*
Male age (and age range)	61 (39–88)	58.5 (41–74)	*p* = 0.013
Female age (and age range)	67 (47–92)	62.5 (51–82)	NS
Arrival time			
early	138 (67.6%)	35 (56.5%)	NS
late	66 (32.4%)	27 (43.5%)	
Peak troponin (mg/L, mean ± sd)			
All cases	1.02 ± 1.74	1.15 ± 1.6	*p* = 0.049
Fatal outcomes only	2.2 ± 3.23	4.05 ± 3.52	NS
Number of fatal outcomes	7 (3.4%)	5 (8.1%)	NS

Note: NS, not significant.

Out of 266 patients, 65% arrived within 120 min, and only 35% later than 120 min after symptom onset. There was no significant difference in the early arrival between diabetic and non-diabetic patients (χ^2^; *p* = 0.105). The peak cardiac troponine T (cTnT) was significantly higher in diabetic compared to non-diabetic patients independently of the time of arrival (*u*-test; *p* = 0.049), but there was no difference in cTnT levels in both groups with fatal outcome. Diabetic patients had a higher early, in-hospital mortality rate *vs.* non-diabetic controls (data not adjusted) but this difference was only numerical and not statistically significant. Early arrivals in general had a lower chance of lethal outcome (χ^2^; *p* = 0.023) (Table 1).

Differences in pain scale score within early- and late-arrival groups are summarized in Table 2. About three fourths of all early arrivals reported their pain as “moderate” or “severe” compared to the late arrival group (χ^2^; *p* < 0.01). However, there was a significantly higher percentage of patients within the non-diabetic group in comparison with diabetics, reporting pain of a moderate to severe degree (χ^2^; *p* < 0.01). Interestingly, the majority of the early arrived diabetic patients experienced “slight” or almost “no pain” at all compared to the non-diabetic group (χ^2^; *p* < 0.01).

**Table 2:** Differences in pain scale score within early- and late-arrival groups.

Pain Scale Score	Early Arrival (*N* = 173; 65%)			Late Arrival (*N* = 93; 35%)		
	Non-Diabetic	Diabetic	Total	Non-Diabetic	Diabetic	Total
No pain and slight pain	28 (20.3%) ^*^	18 (51.4%) ^*^	46 (26.6%)	15 (22.7%)	23 (24.7%)	38 (40.9%)
Moderate and severe pain	110 (79.7%) ^*^	17 (48.6%) ^*^	117 (73.4%) ^*^	51 (77.3%) ^*^	4 (14.8%) ^*^	55 (59.1%) ^*^

Notes: ^*^ *p* < 0.01.

Early arrival was significantly associated with pressing pain as the leading symptom (χ^2^; *p* = 0.036), while late arrival was strongly associated with shortness of breath (χ^2^; *p* = 0.011) (Table 3). Regardless of the in-hospital arrival time, pain quality between non-diabetic and diabetic groups was significantly different in categories of crushing (χ^2^; *p* = 0.011) and burning (χ^2^; *p* = 0.001) both appearing more often in non-diabetic group, while shortness of breath was more common among diabetic patients (χ^2^; *p* = 0.005). Multiple pain qualities were more prevalent among non-diabetic patients (*u*-test; *p* = 0.003). The only reliably present symptom (>50%) in both non-diabetic and diabetic early arrivals was tugging pain.

**Table 3:** Pain quality and troponin levels. Differences within early- and late-arrival groups.

Pain Quality and Troponin	Early Arrival (*N* = 173; 65%)			Late Arrival (*N* = 93; 35%)		
	Non-Diabetic	Diabetic	*p* value	Non-Diabetic	Diabetic	*p* value
Tugging	81 (58.7%) ^*^	14 (40.0%) ^*^	NS	40 (60.6%)	14 (51.9%)	NS
Crushing	66 (47.8%)	12 (34.3%)	NS	31 (47%) ^†^	4 (14.8%) ^†^	NS
Burning	22 (15.9%) ^*^	1 (2.9%) ^*^	NS	8 (12.1%)	0	NS
Pressing	87 (63%)	18 (51.4%)	*p* = 0.036	32 (48.5%)	12 (44.4%)	NS
Sh. of breath	16 (11.6%) ^†^	13 (37.1%) ^†^	NS	18 (27.3%)	10 (37%)	*p* = 0.011
Troponin	0.81 ± 1.7	0.96 ± 1.22	NS	1.45 ± 1.74	1.39 ± 1.99	*p* = 0.049

Notes: ^*^ *p* < 0.05; **^†^** *p* < 0.01; NS, not significant.

When analyzing data according to the time of arrival, a peak cTnT levels were higher in diabetics in the early arrival group (*u*-test; *p* = 0.008) and were associated with late hospital arrivals (χ^2^; *p* = 0.049) (Table 3).

The most frequent intervention in all patients was single-vessel PCI, followed by 2-vessel PCI. PCI failure was not registered among those patients. Emergency CABG was performed in 10 patients (Table 4). The most frequently affected coronary artery was the left coronary artery (LAD) in both groups, followed by the circumflex artery (Cx), and the right coronary artery (RCA) (Table S1).

**Table 4:** Difference in intervention types and mortality in patients clustered according to arrival time and presence of diabetes.

Type of Intervention and Mortality	Early Arrival		Late Arrival	
	Non-Diabetic	Diabetic	Non-Diabetic	Diabetic
no data	3 (2.1%)	3 (8.6%)	0	1 (1.1%)
no stenosis	2 (1.4%)	0	1 (1.5%)	0
PCI, 1 vessel	112 (81.2%)	24 (68.6%)	56 (84.8%) ^*^	12 (44.4%) ^*^
PCI, 2+ vessels	20 (14.4%)	5 (14.1%)	3 (4.5%) ^*^	5 (18.5%) ^*^
CABG	2 (1.4%)	1 (2.9%)	1 (1.5%) ^*^	6 (22.2%) ^*^
fatal outcome	2 (1.4%)	2 (5.7%)	5 (7.6%)	3 (11.1%)

Notes: ^*^ *p* < 0.05; PCI, primary coronary intervention; CABG, coronary artery bypass graft.

A statistically significant difference between diabetic and non-diabetic group was most prominent in the late arrival group since diabetic patients were more likely to suffer from a multi-vessel CAD and therefore underwent a CABG procedure more often (χ^2^; *p* < 0.05), while a single vessel PCI was more frequent in the non-diabetic group of patients (χ^2^; *p* < 0.05) (Table 4).

Duration of hospitalization was not significantly different between non-diabetic and diabetic patients. Patients who underwent CABG because of their ineligibility for PCI were hospitalized one day longer on average (Table S1). The type of antidiabetic therapy (oral *vs.* insulin) had no appreciable influence on number of vessels involved in the intervention, neither had a correlation with any other parameter analyzed in this study.

## 3. Discussion

It is well known that early revascularization in ACS, particularly in STEMI, significantly improves short and long term survival and the remaining myocardial function [11,12,13]. The aim of our study was to analyze whether a potentially worse clinical outcome of STEMI diabetic patients might result from their later hospitalization and differences in pain quality due to impaired pain perception.

The first parameter analyzed was the arrival to the hospital of diabetic and non-diabetic STEMI patients. Surprisingly, contrary to our hypothesis the results have shown that there was no statistically significant difference between number of diabetic and non-diabetic STEMI patients arriving at the hospital within 120 min after symptoms onset (“early”). However, while the majority of non-diabetic STEMI patients that arrived early experienced “moderate” and “severe” chest pain, even 51.7% of diabetic patients experienced “slight” or “no pain” which seemed equally indicative of serious disease as severe pain in non-diabetics. There were several important differences in pain perception between non-diabetic and diabetic patients. Analysis of individual pain qualities within the early arrival group showed a significantly higher frequency of anginal symptoms among non-diabetics and shortness of breath among diabetic patients. Shortness of breath was consistently more often reported in diabetics and that symptom seemed to be the main reason of their early hospital arrival, despite the lower intensity of pain or no pain at all. Sensations of distinct pain, like stabbing and burning, were under-reported by diabetic patients. Obviously, other atypical symptoms were incentive enough for a visit to the emergency department in patients with diabetes. This is consistent with previous studies which have shown that in the presence of myocardial ischemia, diabetic patients reported angina less frequently than non-diabetic patients and shortness of breath may be the only symptom of ischemia [14]. In the study from Zellweger *et al.* [15] only 45% of diabetic patients with myocardial perfusion SPECT (MPS) evidence of CAD had chest pain, whereas 11% reported shortness of breath as the only symptom. This was observed in older patients and in patients with several risk factors, such as peripheral arterial vascular disease, retinopathy, microalbuminuria and autonomic neuropathy [16]. Shortness of breath as angina equivalent could be associated with poorer left ventricular function and outcomes [15], although all our patients with that symptom had normal chest X-ray findings which excluded acute cardiac failure. The statistical significance of this influence is impossible to reliably quantify due to a small number of patients in our study, but seems like a serious candidate for future studies.

In addition, multi-vessel disease was more common in diabetic patients. This was expected and is probably due to atherosclerotic lesions being more severe, diffuse, usually located on small blood vessels and very often not amenable to dilatation due to autonomic neuropathy and silent ischemia in diabetic patients [10,17]. That was a probable cause of an increased frequency of coronary artery bypass procedures in diabetics, which in turn necessitated a slightly longer hospital stay and slower recovery which emphasize the relevance of silent CAD in diabetic patients and, hence, the importance of detecting and screening of silent CAD (*i.e.*, by MPS). This difference was especially prominent in the late arrival group meaning that high risk diabetic patients with large areas of ischemia and developed neuropathy did reach hospital later than desirable. Obviously, the disease itself with its risk factors and complications may dictate a worse outcome despite our finding of an earlier than anticipated arrival of diabetic STEMI patients [18,19,20,21,22,23,24,25,26,27].

Peak cTnT levels were significantly higher in diabetic than non-diabetic patients, and were particularly high in diabetes patients within the early arrival group which corresponds to larger areas of damage and multi vessel disease as was previously demonstrated [15]. Late arrivals in general were associated with higher cTnT levels as was expected.

However, difference in fatal outcome was only numerical and not statistically significant between diabetic and non-diabetic patients. This could be explained that in the present study there was no difference in the arrival times between those two groups and revascularization was performed in timely manner. Furthermore, only in-hospital mortality was taken into consideration, not long-term outcomes.

Incidence of silent ischemia in diabetic patients is increased in various clinical settings and in the presence of multiple risk factors [14,16]. The Croatian population has high CV risk and the incidence of STEMI is still increasing, with reimbursement restrictions for some drugs and/or preventive measures, e.g., statins. Considering the prevalence of diabetes in Croatia of 8.9% [28] and a higher incidence of ACS in diabetic patients [2,7,28] a significant percentage of diabetic patients is expected to have unrecognized episodes of myocardial infarction never reaching the hospital. Due to a lack of systematic data on incidence of type 2 diabetes in Croatia, it is impossible to estimate the number of unrecognized episodes of ACS.

The present study has several limitations. The patient population was relatively small and recruited exclusively from a west region of Croatia which could explain absence of statistical significance in the arrival time as well as in in-hospital mortality rates between two groups. Moreover, because this was an observational study, long term outcome and prognosis in relation to specific time of arrival, type of pain and presence of diabetes remain unknown. Thus, a large-scale long-term prospective study is needed to confirm the present results and determine the relationship between hospital arrival time and pain quality in diabetic patients and the prognosis of obstructive CAD.

## 4. Materials and Methods

### 4.1. Study Design

This observational cross-sectional study complied with the Declaration of Helsinki and was approved by the ethics committee of County Hospital Čakovec. A total of 266 patients with the first presentation of acute STEMI, diagnosed by clinical symptoms, ECG criteria and elevated cTnT according to the American College of Cardiology [29], examined between January 2011 and December 2012 in the Emergency room (ER) of the County Hospital Čakovec were included in the study. All relevant medical data including concomitant medication and medical history were obtained from hospital electronic medical records and patients with previously diagnosed diabetes type 2 formed a “diabetic” group (62), while the other STEMI patients formed a “non-diabetic” group (204). Informed consent was obtained from all patients included in the study.

### 4.2. Patients

The County Hospital Čakovec, Medjimurje region, Republic of Croatia, has been involved in the Croatian 24/7 primary percutaneous coronary intervention (PCI) network for treating patients with ST-elevation myocardial infarction (STEMI) since 2005. Patients from Medjimurje are being transported to the referral PCI center in Zagreb in a way that all eligible STEMI patients are transported to the tertiary center as soon as possible *i.e.*, preferably within 2 h after onset of symptoms, but also later on, usually within 12 h, depending on the pain-to-first-medical-contact time.

Length of the hospitalization, peak Troponin T (TnT) concentrations, type of intervention (PCI or CABG) and a number of significantly occluded coronary arteries according to coronary angiography findings were acquired from hospital electronic medical records. Chest X-ray was performed to exclude dyspnea as a result of acute cardiac failure. For the purpose of this study, a fatal outcome was defined as a death within a scope of an index hospitalization (not only in the 24-hour period).

Pain perception was graded using a modified McGill short form questionnaire, giving each participant a pain score between 0 (no pain) and 3 (severe pain). The quality of pain among the 6 offered categories (stabbing, tugging, crushing, cutting, burning, and pressing) and shortness of breath was noted and subsequently evaluated. The questionnaire was part of the clinical examination. It was completed and evaluated by the research team. Arrival time was rated as either “early” (within 120 min of symptom onset) or “late” (more than 120 min after symptom onset).

### 4.3. Statistical Analysis

Results were analysed using descriptive statistical analysis. Significance was declared at a two-sided 0.05 level, unless otherwise specified. Statistical analyses were performed using SPSS software for Windows (version 15.0, SPSS Inc., Chicago, IL, USA).

Since samples in our study were relatively small and an asymmetric distribution of the analysed variables was present the difference between the two independent variables was analysed by non parametric analysis using chi square and Mann-Whitney test (used for the analysis of sex and age distribution and troponin levels between two groups). Chi square test was also used to estimate the magnitude of the association between variables of interest. Calculated mortality rates were unadjusted 

## 5. Conclusions

In conclusion, the lack of difference in the arrival times between two groups, despite the tendency of less typical clinical presentation in diabetic patients, might be a result of a comprehensive health education for diabetic patients in Međimurje Region, Croatia. It seems that although diabetic STEMI patients have poorer prognosis in general, with well organized care like primary PCI Network program we can significantly reduce CV mortality. Since neuropathy and other changes in pain perception may be indicative for worse outcome in diabetic patients, an additional education should result in their increased awareness of possibility of getting ACS, even at younger age and/or with mild or atypical clinical presentation.

## Supplementary Files

Supplementary File 1

## References

[1] Webster M.W., Scott R.S. (1997). What cardiologists need to know about diabetes. Lancet.

[2] Haffner S.M., Lehto S., Ronnemaa T., Pyörälä K., Laakso M. (1998). Mortality from coronary heart disease in subjects with type 2 diabetes and in non-diabetic subjects with and without prior myocardial infarction. N. Engl. J. Med..

[3] Luscher T.F., Creager M.A., Beckman J.A., Cosentino F. (2003). Diabetes and vascular disease: Pathophysiology, clinical consequences, and medical therapy: Part II. Circulation.

[4] Malmberg K., Yusuf S., Gerstein H.C., Brown J., Zhao F., Hunt D., Piegas L., Calvin J., Keltai M., Budaj A. (2000). Impact of diabetes on long-term prognosis in patients with unstable angina and non-Q-wave myocardial infarction: Results of the OASIS (Organization to Assess Strategies for Ischemic Syndromes) Registry. Circulation.

[5] Brogan G.X., Peterson E.D., Mulgund J., Bhatt D.L., Ohman E.M., Gibler W.B., Pollack C.V., Farkouh M.E., Roe M.T. (2006). Treatment disparities in the care of patients with and without diabetes presenting with non-STsegment elevation acute coronary syndromes. Diabetes Care.

[6] Roffi M., Topol E.J. (2004). Percutaneous coronary intervention in diabetic patients with non-ST-segment elevation acute coronary syndromes. Eur. Heart J..

[7] Božikov V., Rahelić D., Dusper B. (2009). Diabetes and acute coronary syndrome. Acta Med. Croatica.

[8] Creager M.A., Luscher T.F., Cosentino F., Beckman J.A. (2003). Diabetes and vascular disease: Pathophysiology, clinical consequences, and medical therapy: Part I. Circulation.

[9] Stuckey T.D., Stone G.W., Cox D.A., Tcheng J.E., Garcia E., Carroll J., Guagliumi G., Rutherford B.D., Griffin J.J., Turco M. (2005). Impact of stenting and abciximab in patients with diabetes mellitus undergoing primary angioplasty in acute myocardial infarction (the CADILLAC trial). Am. J. Cardiol..

[10] Tbibiazar R., Edelman S.V. (2003). Silent ischaemia in people with diabetes: A condition that must be heard. Clin. Diabetes..

[11] Van de Werf F., Bax J., Betriu A., Blomstrom-Lundqvist C., Crea F., Falk V., Filippatos G., Fox K., Huber K., Kastrati A. (2008). Management of acute myocardial infarction in patients presenting with persistent ST-segment elevation: The task force on the management of ST-segment elevation acute myocardial infarction of the European society of cardiology. Eur. Heart J..

[12] Thygesen K., Alpert J.S., White H.D. (2007). Joint ESC/ACCF/AHA/WHF Task Force for the redefinition of myocardial infarction. Universal definition of myocardial infarction. Eur. Heart J..

[13] Kolh P., Wijns W., Danchin N., Di Mario C., Falk V., Folliguet T., Garg S., Task Force on Myocardial Revascularization of the European Society of Cardiology (ESC), The European Association for Cardio-Thoracic Surgery (EACTS), European Association for Percutaneous Cardiovascular Interventions (EAPCI) (2010). Guidelines on myocardial revascularization. Eur. Heart J..

[14] Kannel W.B., Dawber T.R., Kagan A., Revotskie N., Stokes J. (1961). Factors of risk in the development of coronary heart disease—Six years follow-up experence: The Framingham Study. Ann. Intern. Med..

[15] Zellweger M.J., Hachamovitch R., Kang X., Hayes S.W., Friedman J.D., Germano G., Pfisterer M.E., Berman D.S. (2004). Prognostic relevance of symptoms *versus* objective evidence of coronary artery disease in diabetic patients. Eur. Heart J..

[16] Rutter M.K., McComb J.M., Brady S., Marshall S.M. (1999). Detection of silent myocardial ischemia and microalbuminuria in asymptomatic subjects with non-insulin dependent diabetes mellitus. Am. J. Cardiol..

[17] Bolland L.L., Folson A.R., Sorlie P.D., Taylor H.A., Rosamond W.D., Chambless L.E., Cooper L.S. (2002). Occurence of unrecognized myocardial infarction in subjects aged 45 to 65 years (the ARIC study). Am. J. Cardiol..

[18] Bax J.J., Young L.H., Frye R.L., Bonow R.O., Steinberg H.O., Barrett E.J., ADA (2007). Screening for coronary ARTERY DISEASE in patients with diabetes. Diabetes Care.

[19] Antman E.M., Anbe D.T., Armstrong P.W., Bates E.R., Green L.A., Hand M., Hochman J.S., Krumholz H.M., Kushner F.G., Lamas G.A. (2004). ACC/AHA guidelines for the management of patients with ST-Elevation myocardial infarction—Executive summary: A report of the American College of Cardiology/American Heart Association Task Force on Practice Guidelines. Circulation.

[20] Boersma E. (2006). Does time matter? A pooled analysis of randomized clinical trials comparing primary percutaneous coronary intervention and in-hospital fibrinolysis in acute myocardial infarction patients. Eur. Heart J..

[21] Gierlotka M., Gasior M., Wilczek K., Hawranek M., Szkodzinski J., Paczek P., Lekston A., Kalarus Z., Zembala M., Polonski L. (2011). Reperfusion by primary percutaneous coronary intervention in patients with ST-segment elevation myocardial infarction within 12 to 24 hours of the onset of symptoms (from a prospective national observational study [PL-ACS]). Am. J. Cardiol..

[22] Zijlstra F., Hoorntje J.C., de Boer M.J., Reiffers S., Miedema K., Ottervanger J.P., van’t Hof A.W., Suryapranata H. (1999). Long-term benefit of primary angioplasty as comparedwith thrombolytic therapy for acute myocardial infarction. N. Engl. J. Med..

[23] Poole C.D., Halcox J.P., Jenkins-Jones S., Carr E.S.M., Schifflers M.G., Ray K.K., Currie C.J. (2013). Omega-3 fatty acids and mortality outcome in patients with and without type 2 diabetes after myocardial infarction: A retrospective, matched-cohort study. Clin. Ther..

[24] Keeley E.C., Boura J.A., Grines C.L. (2003). Primary angioplasty *vs.* intravenous thrombolytic therapy for acute myocardial infarction: A quantitative review of 23 randomized trials. Lancet.

[25] Widimsky P., Budesinsky T., Vorac D., Groch L., Zelízko M., Aschermann M., Branny M., St’ásek J., Formánek P., “PRAGUE” Study Group Investigators (2003). Long distance transport for primary angioplasty *vs.* immediate thrombolysis in acute myocardial infarction. Final results of the randomized national multicentre trial—PRAGUE-2. Eur. Heart J..

[26] Dagenais G.R., Lu J., Faxon D.P., Bogaty P., Adler D., Fuentes F., Escobedo J., Krishnaswami A., Slater J., Frye R.L. (2013). Prognostic impact of the presence and absence of angina on mortality and cardiovascular outcomes in patients with type 2 diabetes and stable coronary artery disease: Results from the BARI 2D (Bypass Angioplasty Revascularization Investigation 2 Diabetes) trial. J. Am. Coll. Cardiol..

[27] Young L.H., Wackers F.J., Chyun D.A., Davey J.A., Barrett E.J., Taillefer R., Heller G.V., Iskandrian A.E., Wittlin S.D., Filipchuk N. (2009). Cardiac outcomes after screening for asymptomatic coronary artery disease in patients with type 2 diabetes: The DIAD study: A randomized controlled trial. JAMA.

[28] Poljičanin T., Šekerija M., Metelko Ž. (2011). Šećerna bolest epidemiološko stanje i javno zdravstvene aktivnosti u Hrvatskoj. HČJZ.

[29] Cannon C.P., Battler A., Brindis R.G., Cox J.L., Ellis S.G., Every N.R., Flaherty J.T., Harrington R.A., Krumholz H.M., Simoons M.L. (2001). American College of Cardiology key data elements and definitions for measuring the clinical management and outcomes of patients with acute coronary syndromes. J. Am. Coll. Cardiol..

