# Supplementary material for: Comparison of the Hospital Arrival Time and Differences in Pain Quality between Diabetic and Non-Diabetic STEMI Patients
Source: Int J Environ Res Public Health. 2015 Jan 27;12(2):1387–96. doi: 10.3390/ijerph120201387 (PMC4344672; doi:10.3390/ijerph120201387)
Supplement: Supplementary File 1 [file ijerph-12-01387-s001.pdf]

Supplementary Information

Comparison of the Hospital Arrival Time and Differences in Pain Quality between Diabetic and Non-Diabetic STEMI Patients

Table S1. Frequency of affected coronary arteries and duration of hospitalization in diabetic and non diabetic group of patients.

	**Non-Diabetic**	**Diabetic**	***p* value**
LAD (%)	53	39	NS
Cx (%)	26	34	NS
RCA (%)	21	25	NS
Duration of hospitalization (days)	7 ± 2.9	7.62 ± 3.5	NS

Notes: LAD, left coronary artery; Cx, circumflex artery; RCA, right coronary artery; NS, not significant.

© 2015 by the authors; licensee MDPI, Basel, Switzerland. This article is an open access article distributed under the terms and conditions of the Creative Commons Attribution license (http://creativecommons.org/licenses/by/4.0/).
